# Talking hands: tongue motor excitability during observation of hand gestures associated with words

**DOI:** 10.3389/fnhum.2014.00767

**Published:** 2014-09-30

**Authors:** Naeem Komeilipoor, Carmelo Mario Vicario, Andreas Daffertshofer, Paola Cesari

**Affiliations:** ^1^Department of Neurological and Movement Sciences, University of VeronaVerona, Italy; ^2^MOVE Research Institute Amsterdam, VU University AmsterdamAmsterdam, Netherlands; ^3^School of Psychology, Bangor UniversityBangor, UK

**Keywords:** transcranial magnetic stimulation, tongue motor excitability, speech perception, gesture perception, sign language

## Abstract

Perception of speech and gestures engage common brain areas. Neural regions involved in speech perception overlap with those involved in speech production in an articulator-specific manner. Yet, it is unclear whether motor cortex also has a role in processing communicative actions like gesture and sign language. We asked whether the mere observation of hand gestures, paired and not paired with words, may result in changes in the excitability of the hand and tongue areas of motor cortex. Using single-pulse transcranial magnetic stimulation (TMS), we measured the motor excitability in tongue and hand areas of left primary motor cortex, while participants viewed video sequences of bimanual hand movements associated or not-associated with nouns. We found higher motor excitability in the tongue area during the presentation of meaningful gestures (noun-associated) as opposed to meaningless ones, while the excitability of hand motor area was not differentially affected by gesture observation. Our results let us argue that the observation of gestures associated with a word results in activation of articulatory motor network accompanying speech production.

## Introduction

The processes underlying sign and spoken language perception are known to involve overlapping neural populations. Apparently linguistic information conveyed through gestures and sounds is processed in similar ways (Damasio et al., 1986; Hickok et al., 1996; Neville, 1998; MacSweeney et al., 2002; Newman et al., 2002; Xu et al., 2009; Straube et al., 2012, 2013). This overlap let McNeill (1996) speculate about a unified communication system.

It has been proposed that the evolutionary transition from gesticulation to speech has been mediated by the mirror neuron system, which is believed to underlie the understanding of others’ actions and intentions (Rizzolatti and Arbib, 1998). Interestingly, mirror neurons have first been discovered in monkey area F5 that is considered homolog to human area 44 (Broca’s area), which hosts speech production (Rizzolatti et al., 1996, 2002; Kohler et al., 2002). Nonetheless, no proper evidence supporting the evolution of language from gesture has emerged. According to this idea, vocal communication has become more and more autonomous at the expense of gestures that gradually lost their importance. In *the* *motor theory of speech perception*, Liberman and colleagues have already proposed the motor system to be involved in sensory perception (Liberman et al., 1967; Liberman and Mattingly, 1989). Hence, listeners may perceive spoken language by generating forward models in the motor system by activating articulatory phonetic gestures used to produce acoustic speech signals.

Imaging and transcranial magnetic stimulation (TMS) experiments revealed that speech perception triggers activity in brain areas that are involved in speech production in a somatotopic manner (Fadiga et al., 2002; Watkins et al., 2003; Pulvermüller et al., 2006; D’Ausilio et al., 2014; Möttönen et al., 2014). Repetitive TMS over the left premotor or primary motor cortex causes the capacity of phonetic discrimination to be significantly reduced (Meister et al., 2007; Möttönen and Watkins, 2009; Sato et al., 2009; Möttönen et al., 2014), indicating a causal relationship between the motor system and speech perception. Neural controllers of the articulator’s movement seemingly contribute to both production and perception of speech. Nevertheless it has been argued that the activation of motor areas during listening to speech is neither essential in speech perception nor does it reflect phonetic processing of the speech signal as suggested in motor theory of speech perception (Scott et al., 2009). Evidence from functional lesion studies also supports the idea that involvement of motor areas during speech production does not necessarily contribute to speech perception (for review see, e.g., Hickok and Poeppel, 2000). Scott et al. (2009) argued that several different linguistic functions could be served by motor cortex during speech perception, including a specific role in sensorimotor processing in conversation. But is motor cortex activated differently during the observation of communicative actions such as gesture and sign language?

Recently, Möttönen et al. (2010) reported that motor evoked potentials (MEPs) elicited by stimulating the hand representation in the primary motor cortex (M1) did not differ when participants observed signs with known vs. signs with unknown meanings. If M1 hand area seems insensitive to the distinction between action associated and not associated with words, then other regions in M1 like the tongue or lip areas might be better candidates for this (cf. Fadiga et al., 2002; Watkins et al., 2003; Roy et al., 2008; Sato et al., 2010; D’Ausilio et al., 2014). It remains unclear, however, whether the motor representations of tongue and lips are capable of distinguishing between those actions that symbolically represents words (e.g., an object or a state) and those that do not.

In this study we investigated whether observation of newly learned hand gestures paired and not paired with words may result in changes in the excitability of the hand and tongue areas of motor cortex. We studied MEPs recorded from tongue and hand muscles in a group of healthy Italian participants who had been taught some signs in American Sign Language (ASL). Participants were asked to observe signs associated and not associated with words, i.e., trained and untrained signs. We first trained participants to learn the associated words for several signs (through visual presentation of signs with the associated words as subtitle), while the associated words for the other half of the signs were not taught (the signs presented without subtitles). To ensure that all participants learned the associated words, they underwent a testing session during which participants were observing the video of all the signs but this time without subtitles. They were asked to choose corresponding words for the observed signs among four possible alternatives displayed on the screen. Finally, participants underwent a TMS session, during which we measured the motor excitability in tongue and hand areas of left primary motor cortex while participants were observing the stimuli. We expected the observation of hand gestures alone would lead to similar excitability of hand motor representation, regardless of whether they represent a word or not. We also expected that only the observation of hand gestures associated with words would modulate the excitability of the tongue motor representation.

## Materials and methods

### Experimental procedure

The experiment was designed as a 2 × 2 repeated measurement with two sign types (i.e., meaningful and meaningless indicating hand movements associated and not-associated with words, respectively) and two muscles (tongue and hand). During the experiment TMS-induced MEPs were recorded from tongue and hand muscles while participants observed video sequences of hand movements associated or not-associated with nouns. For each experimental condition 18 MEPs were recorded. The experiment was divided into three sessions: training, test, and TMS.

### Participants

Ten non-signer adult, native Italian speakers (5 females; 23.5 ± 2.6) participated in the study. All were right-handed and had normal or corrected-to-normal vision with no history of speaking or hearing disorders. None of the participants were experienced in ASL. The experimental protocol had been approved by the members of the Ethics Committee of the Department of Neurological, Neuropsychological, Morphological and Movement Sciences of the University of Verona. All participants provided their informed consent prior to entering the study, which had been approved by the institutional review board.

### Stimuli

**Figure 1:**
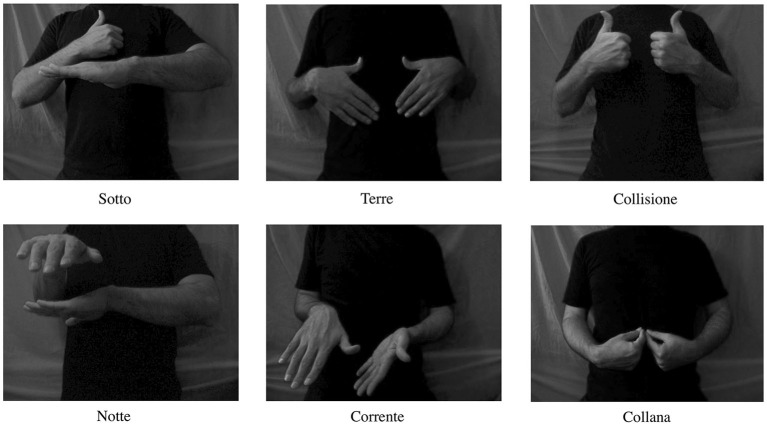
**Illustration of the digital video clips presented to the subjects**. All video clips had duration of 3 s.

Stimuli consisted of six short (duration 3 s) black and white videos depicting hands performing bimanual movements (the actor’s hands and trunk was presented against a gray background). The hand movements were signs in ASL, which were not related in movement structure to any Italian symbolic gestures. Moreover, ASL and not the Italian one was chosen to rule out the contingency of participants’ familiarity with the signs used (having seen the signs and learned their related meanings). All the signs were nouns or adverbs (Necklace, Night, Land, Collision, Below, Current) consisting of double consonants “rr” “ll” “tt” in their Italian translation (Collana, Notte, Terre, Collisione, Sotto, Corrente), which require strong tongue mobilization for proper pronunciation; see Figure 1. The signs were chosen to share the following features: (1) contraction and visibility of the right hand first dorsal interosseous (FDI) muscle in the videos; (2) having associated words that require strong tongue mobilization when pronounced. The FDI muscle was visible and contracted in all the videos to reduce the variability among stimuli because previous studies showed that action observation under different circumstances may lead to modulation of corticospinal motor excitability (for a review, see Rizzolatti and Craighero, 2004). Further, to reduce the variability amongst stimuli concerning the associated words, which would share an element like the visibility and contraction of FDI muscle, we used words with double consonants. These words require strong tongue mobilization when produced, which has already been shown to modulate tongue motor excitability when listening to (Fadiga et al., 2002).

### Training session

Before training participants were informed that they were going to view six videos of various hand movements each repeated ten times, three of which had related-word in the form of a subtitle and three did not. We restricted the study to six stimuli to ensure that all participants could readily learn the three associated words. Participants were instructed to memorize the association gesture-word from each of the three videos with the subtitles. One group learned the associated gesture-words of (Collana, Notte, Terre) and the other one learned (Collisione, Sotto, Corrente). The training was set up as follows: at first a screen-centered fixation cross was displayed for 1000 ms; subsequently, video stimuli (three with subtitles and three without) were presented in random order for 3 s. Participants were asked to be silent during the entire experiment. To test whether the participants learned the meaning of the signs, they underwent a test session after training.

### Test session

During the test session the videos of the training session were presented at random without subtitles, each repeated ten times. After each stimulus presentation, participants were asked to choose the corresponding word for the observed signs among four possible alternatives displayed on the screen until participants choose the correct answer by clicking the right mouse button with the index finger of the left hand. The four possible choices were the three learned words plus a question mark indicating “I do not know the answer”. The displays were centered in the four quadrants of the screen. For every answer participants received feedback of correctness (knowledge of results). The feedback for the correct answer was displayed in white on a black background in the center of screen, and the feedback for the incorrect answer was displayed in red on a white background. Stimulus order and target position on the screen were randomized. All the participants accomplished the test session successfully (100% of correct responses) without any errors rendering ongoing ASL learning unlikely.

### TMS/EMG session

#### Procedure

The experiment was designed using the E-Prime 2 (Psychology Software Tools, Inc, USA) software running on a PC computer with a Windows XP operating system to control the stimulus presentation, randomization of trials and to trigger the TMS and EMG recordings. Transcranial magnetic stimulation induced EMG activity was collected from all participants.

During the experiment, the subjects were comfortably seated on an armchair in a dimly-lit room at a distance of 80 cm from a computer screen (Asus, 17”, 60 Hz refresh rate). Each trial started with a fixation cue (the “+” symbol), presented for 1000 ms immediately followed by the stimulus that lasted for 3 s. The left M1 was stimulated via a single-pulse TMS delivered through a figure-of-eight coil at 120% of the individual resting motor threshold (over both tongue and hand motor areas). The TMS pulses were generated randomly within the last 2 s of stimulus presentation (from the beginning of the second to the end of the third second), when in the observed actions the FDI muscle was contracting and the meaning had already been conveyed. This was done to ensure that FDI muscle was clearly observable when the TMS pulses are delivered, and to give the participants more time to recognize the associated word. After each stimulus presentation, participants were asked to choose the corresponding word for the observed signs in a same way they did during the testing session. After pressing the space button to continue, the next stimulus was delivered with an inter stimulus interval of 8 s. Every TMS session took about 15 min and consisted of 36 trials (18 per each condition). The two sessions (tongue and hand stimulation) were carried out on the same day and their order was counter-balanced across participants.

#### Data acquisition

Focal TMS was applied with a 70-mm figure-of-eight coil that was powered by a STM9000 Magnetic Stimulator (ATES Medical Device, IT) producing a maximum output of 2T at the coil surface. Before each session, the coil was moved over the scalp in order to determine the optimal site from which maximal amplitude MEPs were elicited in the tongue and hand muscles separately. The coil was held tangentially to the scalp with the handle pointing 45° away from the nasion–inion line in a posterolateral direction (Mills et al., 1992) to find the FDI representation area. Following the same procedure pursued in a previous work of our group (Vicario et al., 2014), the tongue area was stimulated with the coil handle oriented at 90° directed straight posteriorly.

**Figure 2:**
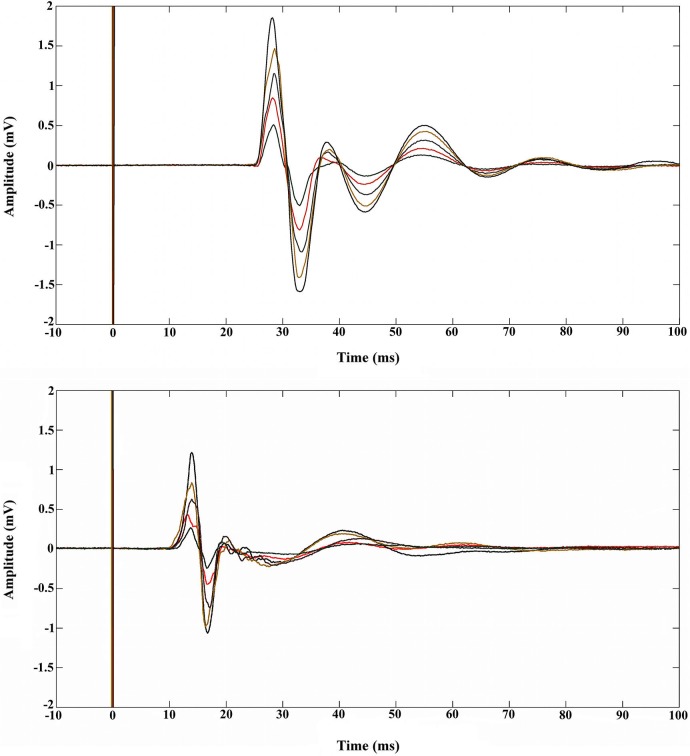
**Examples of five MEPs recorded during rest in the FDI muscle (upper panel) and in the tongue muscle (lower panel)**. The vertical lines at 0 ms indicate the moment at which the single pulse TMS was elicited.

The resting motor threshold of the muscles was determined according to standard methods as the minimal intensity capable of evoking MEPs in 5 out of 10 trials of the relaxed muscles with amplitude of at least 50 μV (Rossini et al., 1994). Bipolar EMG from the tongue muscles were acquired using a pair of Ag-AgCl surface electrodes (Ø 1 cm). The electrodes were pasted on plastic buttons and fixed on a spring of iron zinc. Before recording, electrodes were immersed in a disinfectant solution (Amuchina, sodium hypochlorite 1.1 grams per 100 ml of purified water) for 5 min and rinsed in drinking water. Participants were asked to introduce their tongue within these two electrodes, adjust the spring so that it was perfectly fitting with the tongue, and remain as relaxed as possible for the full duration of the experiment. The ground electrode was placed on the forehead of the participant. In separate sessions, EMG activity was recorded from the FDI muscle of the right hand by placing surface electrodes over the muscle belly (active electrode) and over the tendon of the muscle (reference electrode). The ground electrode was placed over the dorsal part of the elbow. The activity of muscles were registered in separate blocks and counterbalanced across participants. Electromyography signals were band-pass filtered online (20–3000 Hz), amplified (Digitimer, Hertfordshire, England) and sampled at a rate of 5 kHz (CED Micro 1401, Cambridge Electronic Design, Cambridge, England). Motor evoked potentials’ peak-to-peak amplitude (in millivolts) were calculated off-line using Spike 2 (version 6, Cambridge Electronic Design) and stored on a computer. We determined muscle pre-activation through visual inspection and excluded contaminated trials from the analysis (6.3% of trials, See Figure 2 for MEP examples).

## Statistics and results

**Figure 3:**
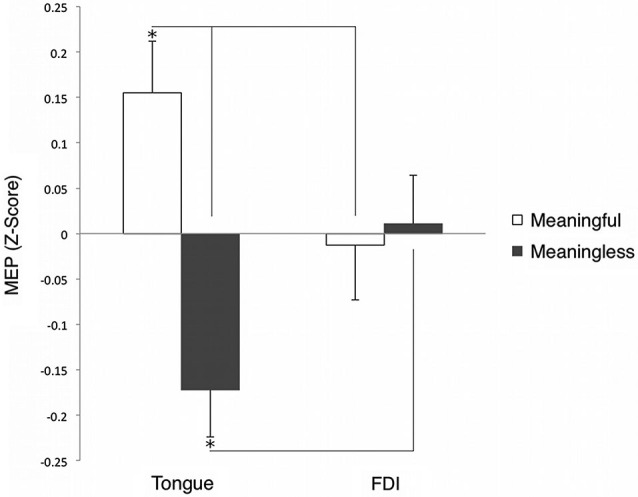
**Grand averaged (*z*-score of the) MEP amplitude of tongue and hand muscles during observation of meaningful and meaningless signs**. *p < 0.05.

Motor evoked potentials’ amplitude values were normalized (*z*-scored) for every subject and muscle. A two-way repeated measures ANOVA was performed with two sign types (meaningful and meaningless) and two muscles (tongue and hand). *Post hoc* comparisons were performed by means of *t*-tests applying a Bonferroni correction for multiple comparisons when required. A partial-eta-squared statistic served as effect size estimate. The interaction between the sign types and muscles was significant; (*F*_(1,9)_ = 7.875, *p* = 0.021, *η*^2^ = 0.46). Tongue cortical excitability was enhanced during the presentation of meaningful (trained) as compared to meaningless (untrained) signs (*p* = 0.02). That is, the presentation of word-associated gestures yielded an increase in tongue MEPs compared to the observation of signs that were not associated with words. The hand MEP *z*-scores did not reveal significant differences between the two types of signs (and, therefore, the mean *z*-scores were close to zero) (*p* > 0.05)^1^. Further *post hoc* analysis (Bonferroni test) indicated that observation of word-associated signs elicited significantly larger MEP amplitudes, relative to meaningless signs on the tongue compared to the FDI muscle (*p* = 0.025). By contrast, meaningless signs were accompanied by relative decrement of MEP amplitudes in *z*-scores on the tongue as compared to the FDI muscle (*p* = 0.019); see Figure 3. Moreover, the raw MEP amplitudes recorded from the hand for each individual participant were greater than those recorded from the tongue muscle (cf. Figure 2). Because MEPs amplitude values were normalized using *z*-scores for each muscle, differences between MEP amplitudes of the two muscles (as shown in the Figure 3) are not necessarily indicative of differences in the magnitude of excitability.

## Discussion

To the best of our knowledge we have provided the first experimental evidence for the modulation of excitability in the tongue area of M1 cortex as a function of observation of word-associated movements. We found the highest cortical excitability in the tongue area during the presentation of word-associated gestures compared with gestures not associated with any words (meaningless). On the contrary, the hand motor area presented the same level of excitability for both type of gestures. Our results are in line with the TMS study by Möttönen et al. (2010) showing that MEPs elicited by the stimulation of the hand representation in the left M1 did not significantly differ when participants observed signs with known vs. signs with unknown meanings. To unravel motor cortex modulation during gesture observation, they recorded TMS-induced MEPs from hand muscles of participants during sign language observation. They also compared the MEPs obtained before and after individuals learned the meanings of the signs presented and found that the excitability of left and right hand representation in M1 was equally lateralized before participants knew that the presented hand movements were signs. By contrast, after learning both known and unknown signs, the motor cortical excitability significantly increased only on the left M1 side, supporting the left hemispheric dominance for language processing (Knecht, 2000). Moreover, it has been suggested that brief inactivation of Broca’s area by use of repetitive TMS affects verbal responses to gesture observation, suggesting the involvement of Broca’s area in the instantaneous control of gestures and word pronunciation (Gentilucci et al., 2006). In addition, the very recent study by Vicario et al. (2013) showed that M1 might be indirectly involved in the mapping process of newly acquired, action-related, categorical associations.

The current work complements these findings and underscores the contribution of tongue but not hand motor area in the processing of communicative hand actions associated with the words. Several TMS studies have demonstrated modulation in the excitability of tongue motor area during speech perception (Fadiga et al., 2002; Watkins et al., 2003; Roy et al., 2008; Sato et al., 2010; D’Ausilio et al., 2014). It has been thoroughly argued that motor activation during speech perception emerges as a result of different task demands or experimental conditions rather than being an essential activity underlying speech perception (for review see, e.g., Lotto et al., 2009; Scott et al., 2009). Moreover, whether articulatory commands activated automatically and involuntarily during speech perception, is still a matter of debate (McGettigan et al., 2010). Here we have shown that it is not the hand but the tongue motor area that is specifically involved during the observation of gestures associated with a word, although individuals were not required to pronounce that word. Note that an additional control condition such as videos showing objects or symbols or fractals associated to specific words would have enabled us to determine whether the excitability in the M1 tongue area was a function of sign language observation or due to the effects of covert speech associated to the observed video. This should be addressed in future work.

Previous TMS studies have reported facilitation of the corticospinal tract excitability during the mere observation of another person’s actions (for a review see Fadiga et al., 2005). The mirror system is active under various circumstances. For instance, somatotopic activation is present in the motor cortex when individuals observed and imagined actions (for a review, see Rizzolatti et al., 1996; Fadiga et al., 1999; Rizzolatti and Craighero, 2004). Even more critical is the mirror system involvement when the actions are not directly visible to the observer but implicitly presented (Bonaiuto et al., 2007). Building on to a vast amount of literature, one may speculate that humans have internal representations of the movements either observed or imagined and that these internal representations resemble very closely the action when it is actually performed. In the present study we aimed for determining whether and how observation of hand gestures linked and not linked to specific words involves an internal motor simulation. We showed that while the observation of hand movements required similar internal motor simulations within the hand area of M1, regardless of whether they are associated with a word or not, only the observation of hand movements associated with words activated the tongue area of M1, indicating an extra level of coding. We here suggest that disentangling word-associated gestures, i.e., meaningful signs vs. meaningless signs, leads to internal simulations within the language motor regions (i.e., tongue).

The so-called gestural-origins theory of speech ascribes a precise role in language evolution to gestures (Corballis, 2003; see also Vicario, 2013 for a recent discussion). It has been suggested that spoken language evolves from an ancient communication system using arm gestures. Gestures of the mouth might have been added to the manual system to form a combined manuofacial gestural system (Corballis, 2003; Gentilucci and Corballis, 2006). Our results may suggest that the perception of sign language might require similar neural activity in speech motor centers as speech perception does. In this sense, our findings contribute to the view that the perception of speech and gesture share common neural substrates. Recent neuroimaging studies have revealed that semantic processing of speech and gestures engages common brain network with a specific involvement of left motor cortex (Xu et al., 2009; Straube et al., 2012, 2013). This is particularly interesting because it implies that motor cortex may be activated in response to language information independently of the communication modality.

Viewing sign language by deaf signers activates the classical language areas (left frontal and temporal areas) similar to the pattern of activity present when hearing participants listen to spoken words (Neville, 1998; MacSweeney et al., 2002; Newman et al., 2002). Damage to the left hemisphere often produces sign language aphasia just like aphasia in spoken language, suggesting the left cerebral hemisphere dominancy for both signed and spoken languages (Damasio et al., 1986; Hickok et al., 1996). Taken together we conclude that the involvement of the tongue region of the primary motor cortex is not merely limited to the perception and production of speech but might rather play a general role in encoding linguistic (maybe related to phonological retrieval) information even during perception of actions paired with words.

## Conflict of interest statement

The authors declare that the research was conducted in the absence of any commercial or financial relationships that could be construed as a potential conflict of interest.

## Footnotes

^1^To test whether this non-significant result was due to a lack of statistical power, we conducted an analysis using G* Power (Faul et al., 2009) with *t*-test as family test and “Means: difference between two dependent means (matched pairs)” as a statistical test and “*A priori*: compute required sample size—given *α*, power, and effect size” as analysis type. The input parameters and their values were set as follows: Tail(s) = Two, Effect size *dz* = 0.3234 (derived from differences in the mean (0.024) and the standard deviation (0.0742)), *α* err prob = 0.05 and Power (1 – *β* err prob) = 0.8. This analysis indicated that the sample size ought to be increased to *N* = 77 for the hand muscle to reach statistical significance at a 0.05 level. It is hence unlikely that the non-significant results found for hand muscle could be attributed to a limited sample size.
